# Analysis of Bitter honey using gas chromatography and Tandem Mass Spectrometry

**DOI:** 10.6026/97320630018196

**Published:** 2022-03-31

**Authors:** Koodathil Joshna, Venkatachalam Gopal, Baskaran Kavitha

**Affiliations:** 1 Department of Pharmacognosy, College of Pharmacy, Mother Theresa Post Graduate and Research Institute of Health Sciences, Gorimedu, Puducherry -605006, India

**Keywords:** Bitter honey, volatile compounds, GCMS/MS

## Abstract

Honey has been consumed by humans since ancient times. Honey contains volatile compounds like aldehyde, alcohol, ketone, hydrocarbon, terpenes, acids, benzene compounds. These compounds represent the fingerprint of monofloral honey there by providing information about the floral and geographical origin of honey. The volatile compounds present in honey not only contribute the aroma but also associated with the therapeutic activities of honey. In the present study, the GCMS/MS analysis of bitter honey was carried out to identify the presence of volatile compounds. This is the first study to determine the volatile compounds from ethyl acetate extract of bitter honey produced in the Nilgiri biosphere. Among the eighteen compounds detected, the majority of the compounds were reported to be therapeutically active. Hence further studies regarding the isolation of these compounds could be beneficial in the treatment of various diseases.

## Background:

Honey has been used in traditional medicine since antiquity [1]. The chemical constituents present in honey mainly depend on the botanical and geographical source of honey [2]. The characteristic aroma of honey is due to the presence of minor volatile components [3]. The volatile compounds present in the honey includes norisoprenoids, terpenes, benzene compounds ketones, aldehydes, acids, alcohol, hydrocarbon, pyran and furan derivatives [4]. Terpenes and their derivative compounds contribute the odor, flavor, and medicinal properties of honey [5]. The volatile compounds present in honey can be considered as a fingerprint of its botanical origin [6]. Honey has been studied for its antibacterial, antioxidant, antidiabetic, antiulcer, and anti-inflammatory activity [7-10]. GCMS is the widely used technique to identify the volatile compounds present in honey [11]. Bitter honey is a different variety of monofloral honey, bitter after taste produced mainly in the Nilgiri biosphere. Syzygiumcumini is the predominant source of bitter honey which the Apisdorsata bees preferred during foraging. To the best of our knowledge, there are no GCMS/MS studies carried out for the determination of volatile compounds in bitter honey. Therefore, it is of interest to report the phytochemical analysis of ethyl extract of bitter honey.

## Materials and methods:

### Collection of bitter honey:

Bitter honey sample was collected from the honey hunters of Nilgiris in the year 2018. The samples were stored in a dry place until further analysis.

### Extraction of bitter honey:

Bitter honey sample was weighed accurately about 10g and transferred into a glass beaker. To this 20ml of distilled water was added and shaken for about ten minutes using a mechanical shaker. The sample was extracted by adding 100 ml of ethyl acetate and 50g of anhydrous sodium sulphate using a homogenizer. The extract was then evaporated to dryness using a rotary evaporator. The evaporated extract was filtered and used for further analysis [12].

### Chromatographic analysis:

The GCMS/MS analysis of bitter honey was performed using a TSQ 9000 Triple Quadrupole GCMS/MS model (Thermo fisher Scientific) equipped with TSQ QUADRPOLE Mass spectrometer detector. The column used was Column trace GOLDTM TG-624,30Mx0.25mm IDx1.4µm df. Helium (1ml/min) was used as a carrier gas and the split ratio was maintained as 10:1. One microliter of the sample was injected into the column and the oven temperature was maintained initially at 110°C for 3.50 minutes. The temperature was increased to 10°C/min in the second stage up to200°Cand finally up to at 280°C at the rate of 5°C/min for about 12minutes. The injector temperature was maintained at 280°C [13].

### Identification of bioactive phytochemical compounds:

MS program: The inlet temperature was set at 290°C and the source temperature was programmed as 250°C. The ionization voltage was taken as 70eV and the mass scan was performed at 50-500amu. The solvent delay was about 0-3.5 minutes and the total MS running time was 40-50 minutes. The software used to handle the mass spectra was X-Caliber. The identification of the volatile compounds in bitter honey was carried out by comparing the retention time of the reference listed in NIST Library version 2011 [14].

## Results and Discussion:

GCMS/MS method of qualitative analysis of compounds present in ethyl acetate extract of bitter honey was successfully determined and the chromatogram was represented in Figure 1(see PDF). The components identified with the retention time, molecular formula, molecular weight, and peak area were represented in Table 1(see PDF). The highest peak area was observed in 3-Buten-2-one,4-(2-hydroxy-2,6,6- trimethyl cyclohexyl) in which studies have reported the antiulcer, antimicrobial, antioxidant, wound healing, and anti-inflammatory activity [15]. 3, 3-Dimethyl-4-(3,3,4,4-tetra methyloxetan-2-ylidene) butan-2-one, anantimicrobial compound was identified in bitter honey with the second-highest peak area [16. Other compounds like 9-Octadecenoic acid (Z)-or oleic acid were reported to have beneficial effects on wound healing, autoimmune diseases, and in the treatment of cancer [17]. Dodecanoic acid or lauric acid identified in bitter honey has been studied for its antimicrobial and antiviral activity [18]. Corymbolone, a compound identified was reported to possess an antiplasmodial effect against Plasmodium falciparum and 3-Buten-2-one, 3-methyl-4-(1, 3, 3- trimethyl-7-oxabicyclo [4.1.0] heptan1-yl)-, a ketone was studied for its reduced blood glucose and increased serum insulin levels in db/db mice [19, 20]. Dasycarpidan-1-methanol, acetate (ester) identified in bitter honey was reported earlier for its antimicrobial activity [21].

## Conclusion:

Studies regarding the volatile compounds in bitter honey were found to be scientifically underexploited. From the results of the present study, it can be concluded that bitter honey is rich in therapeutically active compounds. Hence the dietary intake of bitter honey can be recommended to improve overall health as a prophylactic and as a treatment. Further studies regarding the isolation of volatile compounds from bitter honey will be beneficial to introduce the medicinally important phytochemicals to limelight.
